# Prevalence of Kaposi’s sarcoma-associated herpesvirus in Uygur and Han populations from the Urumqi and Kashgar regions of Xinjiang, China

**DOI:** 10.1007/s12250-017-4049-9

**Published:** 2017-10-25

**Authors:** Jun Zheng, Yang Yang, Meng Cui, Zhan-Jun Shu, Li-Li Han, Zhen-Qiu Liu, Charles Wood, Tiejun Zhang, Yan Zeng

**Affiliations:** 1 Key Laboratory of Xinjiang Endemic and Ethnic Disease & Department of Biochemistry, School of Medicine, Shihezi University, Shihezi, 832000 China; 2 Department of Stomatology, The First Affiliated Hospital, School of Medicine, Shihezi University, Shihezi, 832000 China; 3 Division of AIDS Research, National Traditional Chinese Medicine Clinical Research Bases in Xinjiang, Urumqi, 830000 China; 4 Department of Gynecology, Xinjiang Uygur Autonomous Region People’s Hospital, Urumqi, 830001 China; 5 Department of Epidemiology, School of Public Health, Fudan University, Shanghai, 200032 China; 6 Nebraska Center of Virology and the School of Biological Sciences, University of Nebraska-Lincoln, Lincoln, 68583 USA

**Keywords:** Kaposi’s sarcoma associated herpesvirus (KSHV), prevalence, Uygur ethnicity, Han ethnicity, Xinjiang

## Abstract

Kaposi’s sarcoma-associated herpesvirus (KSHV) is the infectious etiologic agent associated with Kaposi’s sarcoma (KS), primary effusion lymphoma, and multicentric Castleman disease. It has been shown that high KSHV prevalence and high incidence of both classic KS and AIDSassociated KS are found mostly among people of Uygur ethnicity in Xinjiang, while people of Han ethnicity in Xinjiang have a higher KSHV seroprevalence than those of other Han populations in mainland China. However, it is still unclear why there is such geographical and population variation in KSHV distribution in China. In this work, we focused on the populations in the Kashgar region and Urumqi area, where a total of 1294 research subjects were randomly selected to investigate the potential correlation between KSHV prevalence and different ethnicities in endemic areas of Xinjiang, and to determine risk factors that may affect KSHV infection rates or KS incidence. We identified a high seroprevalence of KSHV and high peripheral blood DNA infection in the general Uygur and Han populations in both Urumqi and Kashgar regions of Xinjiang, and determined that advancing age, low education level, and stationary population status affect KSHV infection rates. Further, KSHV-positive Uygur participants were shown to have higher prevalence of neutralizing antibodies and neutralizing antibody titers than KSHV-positive Han participants.

**Electronic Supplementary Material:**

Supplementary material is available for this article at 10.1007/s12250-017-4049-9 and is accessible for authorized users.

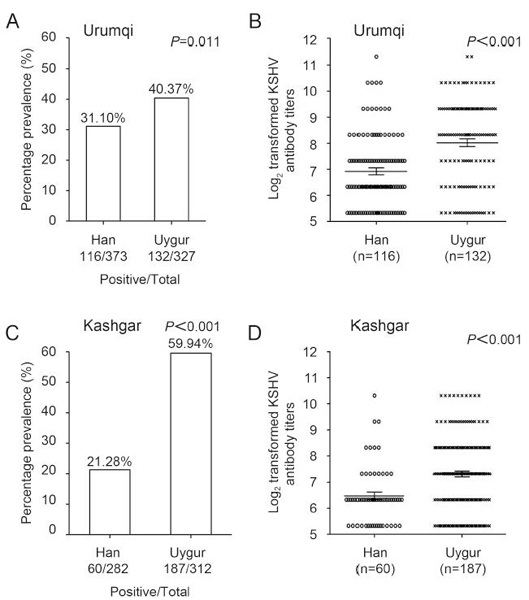

### INTRODUCTION

Kaposi's sarcoma (KS) is a soft-tissue tumor most commonly found in individuals with AIDS or immunodeficiency (Boshoff et al., 2001). It can be classified into four forms based on its clinical and epidemiological patterns: classical, AIDS-related, endemic, and iatrogenic KS (Cook-Mozaffari et al., 1998). Kaposi's sarcoma-associated herpesvirus (KSHV), also known as human herpesvirus 8 (HHV8), was discovered in 1994 and is now known to be the etiologic agent for KS, primary effusion lymphoma, and multicenter Castleman's disease (Cesarman et al., 1995; Chang et al., 1994; Soulier et al., 1995; Whitby et al., 1995).

The prevalence of KSHV infection parallels the incidence of KS. KSHV seroprevalence is generally low to moderate in western countries where KS incidence is low, ranging from 3% to 23% (Ablashi et al., 2002). That being said, KSHV seroprevalence is much higher in the HIV-positive population (Bower et al., 2006). In Africa, where there is a high incidence of endemic KS and an HIV epidemic in the region, KSHV seroprevalence is found to be as high as 50% in a number of African countries (Dedicoat et al., 2003; Minhas et al., 2008). In contrast, data from Asian countries suggest that KSHV seroprevalence is generally low (Ablashi et al., 2002; Fujii et al., 1999). This is consistent with the much lower incidence of KS in the region. In China, KS incidence in low throughout most of the country, although a high incidence of classical KS has been reported in the Xinjiang region (Fu et al., 2009; Wang et al., 2011). Xinjiang is a region of autonomous rule in northwestern China that borders Russia, Kazakhstan, Kyrgyzstan, Tajikistan, Pakistan, Mongolia, Afghanistan, and India. The population in this region is made up of a number of ethnic groups, including Uygur, Han, Kazakh, and Hui. In parallel with the higher incidence of HIV infection, classic KS and AIDS-associated KS are found mostly in those of Uygur and Kazakh ethnicity (Dilnur et al., 2001). KSHV prevalence also varies considerably among geographical regions and populations in the country, and it is associated with the KS incidence. In most provinces of China, KSHV seroprevalence is found to be less than 8% in the general population. However, it is reported to be 19.3% to 46.6% in the general adult population of Xinjiang province, especially among the Uygur and Kazakh populations (Fu et al., 2009; Wang et al., 2011). Also noticeable is that Xinjiang has one of the highest levels of HIV infection in China; prevalence of KSHV among HIV-positive individuals was 48.9% in Xinjiang from 2014 through 2015 (Liu et al., 2017). However, despite the high KSHV prevalence and the high incidence of KS in Xinjiang, it remains unclear why there is such geographical and population variation in KSHV distribution in China.

The Han population in Xinjiang shows a wider distribution, predominantly residing in northern Xinjiang where it accounts for about 70% of the total population in comparison with the Uygur 25%. As the capital city of Xinjiang, Urumqi accommodates a large proportion of the Han population, which amounts to 72% of the total local population and more than 10% of the total Han population in Xinjiang. In addition, a relatively large portion of the Han population in the Urumqi area is transient. Compared with the Han population, the distribution of the Uygur population is relatively concentrated. The Uygur population is mainly distributed in the southern region of Xinjiang, accounting for more than 85% of the total Uygur population in Xinjiang. From a regional point of view, nearly 36% of the total Uygur population in Xinjiang Uygur Autonomous Region live in Kashgar, accounting for 89% of the Kashgar region's total population.

In this work, we focused on the populations in the Kashgar region and the Urumqi area. The primary objective was to investigate the potential correlation between KSHV prevalence and different ethnicities in endemic areas of Xinjiang, and to determine whether environmental factors or population migration affect KSHV infection rates or KS incidence.

### MATERIALS AND METHODS

#### Study participants and sample collection

A total of 1294 research subjects were randomly selected from two regions of Xinjiang between January and December 2015 using a stratified, multistage sampling. Blood samples obtained from the research subjects were divided into two groups. Group 1 included 700 plasma samples and whole blood samples from individuals in Urumqi. Group 2 comprised plasma samples from 594 individuals in Kashgar. Written informed consent was obtained from all participants of the study. Additionally, KSHV-seronegative plasma samples from anonymous healthy blood donors from a local blood bank in Lincoln, NE, USA, were used for assay controls.

Blood samples were collected in EDTA tubes, and plasma was separated. Specimens were coded by a unique identification number. All specimens were stored at –70 °C until testing.

#### KSHV serological test

Two KSHV serological tests were used. First, all plasma samples were diluted 1:40 in PBS and tested using monoclonal-enhanced immunofluorescence assays (mIFAs), as described by Minhas *et al.* (2008). Briefly, BC3 cells were stimulated with tetradecanoyl phorbol acetate, then fixed with 4% paraformaldehyde and incubated with patient plasma. Mouse monoclonal antihuman IgG (CRL-1786; American Type Culture Collection, Manassas, VA, USA)

was used as a secondary antibody and DyLight488-conjugated donkey anti-mouse IgG (Jackson Immuno Research, West Grove, PA, USA) was used as the tertiary antibody. Second, *Spodoptera frugiperda* clone 9, expressing three viral recombinant proteins, ORF73, ORF65, and K8.1, was used (Fu et al., 2009). The procedure was similar to the BC-3 immunofluorescence assay. A sample was considered to be HHV-8-seropositive only if it was positive at a standard serum dilution of 1:40 with both the BC-3 and the *S. frugiperda* clone 9 assay. Each slide was read independently by two experienced laboratory workers. To determine the geometric mean titer (GMT) of KSHV antibody, all positive plasma was diluted further and tested by mIFA to estimate the KSHV antibody titer of each sample.

### DNA extraction and nested PCR for KSHV detection

DNA was extracted from the whole blood samples using a Gentra Puregene Blood kit (Qiagen, Germany) according to manufacturer's protocol. Extracted DNA was subjected to polymerase chain reaction (PCR); the sequences of primers are listed in Supplementary Table 1. PCR-positive samples were analyzed by nested PCR for the presence of KSHV DNA. Each PCR reaction was performed in a total volume of 25 μL, using 0.4 μmol/L primers and a TaKaRa Ex Taq DNA polymerase kit (TaKaRa Biotechnology) according to the manufacturer's protocol, with the exception that 2.5 units enzyme was used. For control *β-actin* reactions, 1 μL genomic DNA was used. For first- and second-round KSHV *ORF26* PCR, 2 μL genomic DNA and 2 μL PCR product were used, respectively. All reactions were performed under the following conditions: 95 °C for 5 min, 35 cycles of 95 °C for 30 sec, 58 °C for 30 sec, 72 °C for 30 sec, and one cycle of 72 °C for 7 min. Samples positive for the *ORF26* gene were then tested for presence of the KSHV *gB* gene by nested PCR amplification. Positive results for DNA detection were defined as those samples positive for both *ORF26* and *gB* genes.

Table 1. Characteristics of the study population

| Characteristic | Group 1 (Urumqi) | Group 2 (Kashgar) |
|---|---|---|
| | n (%) | n (%) |
| Total Number | 700 | 594 |
| **Ethnicity** | | |
| Han | 373 (53.3) | 282 (47.5) |
| Uygur | 327 (46.7) | 312 (52.5) |
| **Gender** | | |
| Male | 364 (52.0) | 244 (41.1) |
| Female | 336 (48.0) | 350 (58.9) |
| Median Age (years) | 43 | 48 |
| **Education** | | |
| Elementary or below | 273 (39.0) | 330 (55.6) |
| Junior high or beyond | 427 (61.0) | 264 (44.4) |
| **Population** | | |
| stationary population | 437 (62.4) | 507 (85.4) |
| immigrated population | 263 (37.6) | 87 (14.6) |

### KSHV neutralization assay

Neutralizing antibodies against KSHV were detected for all seropositive samples by a flow cytometry based neutralization assay as described previously with minor modifications (Kimball et al., 2004). Plasma was diluted and mixed with recombinant KSHV expressing green fluorescent protein (GFP; rKSHV.219) and incubated for 1 h at 37 °C. The virus and plasma mixture was added into a 96-well plate seeded with 293 cells. After incubation for 72 h, green cells were counted by flow cytometry. Results were confirmed by counting at least 100 cells under a fluorescence microscope. A positive neutralizing antibody outcome was defined as ≥ 50% reduction in infectivity as compared with the seronegative control serum. Positive plasma samples were diluted further to determine the neutralizing antibody titer.

### Statistical analysis

Original questionnaire data and laboratory results were entered and managed with EpiData 3.0 (Odense, Denmark), and then transferred to SPSS 17.0 (SPSS Inc., Chicago, IL, USA) for further analysis. Differences in KSHV seroprevalence were measured by chi-square test or Fisher's exact test. The Mann-Whitney *U*-test was used to assess the difference in GMTs of KSHV between Uygur and Han ethnic groups. Correlation analysis was evaluated by Pearson's correlation coefficient. Power and sample size estimation was done using Sample Power 2 (SPSS). GraphPad Prism 5.0 (GraphPad, La Jolla, CA) was used to construct figures. A $P$ value of less than 0.05 was considered statistically significant.

## RESULTS

### Characteristics of study participants

The characteristics of the two groups in the study population (N = 1294) are summarized in Table 1. Group 1 participants (N = 700) aged 16–82 years were recruited from Urumqi. Among them, 373 participants (53.3%) were of Han ethnicity and 327 participants (46.7%) were of Uygur ethnicity. Group 2 participants (N = 594) aged 15–86 years were recruited from Kashgar. comprising 282 participants (47.5%) of Han ethnicity and 312 participants (52.5%) of Uygur ethnicity.

### KSHV seroprevalence and antibody titers in Urumqi and Kashgar regions

The overall KSHV seroprevalence of Uygur participants was 40.4%, which was significantly higher than that of Han ethnicity participants (31.1%) in Urumqi ($P < 0.05$) (Figure 1A). The end-point titer of all KSHV-positive Han and Uygur plasma samples was determined, and we observed that the GMT of Uygur ethnicity individuals was significantly higher than that of the Han ethnicity individuals ($P < 0.001$) (Figure 1B). The KSHV seroprevalence of Uygur participants in Kashgar was 59.9%, which was also significantly higher than that of Han participants (21.3%) ($P < 0.001$) (Figure 1C), and the GMT of all KSHV-positive Uygur ethnicity individuals was significantly higher than that of all KSHV-positive Han ethnicity individuals ($P < 0.001$) (Figure 1D).

### Risk factors associated with KSHV seroprevalence

The association between KSHV seroprevalence and the characteristics of the 1294 participants stratified by geographic region is presented in Table 2. In the Urumqi group of 700 study participants, 116 Han (31.1%) and 132 (40.4%) Uygur were KSHV seropositive. KSHV

Figure 1. KSHV seroprevalence and antibody titers in Han and Uygur groups in the study population of Urumqi and Kashgar, Xinjiang. (A, C) Prevalence of KSHV infection in Han and Uygur populations of Urumqi and Kashgar. (B, D) Comparison of KSHV antibody titers in Han and Uygur populations of Urumqi and Kashgar.

infection was correlated with advancing age ($P = 0.005$) and education level ($P = 0.047$). Interestingly, participants in the stationary population exhibited high serum prevalence of KSHV (38.4%). In the Kashgar group of 594 study participants, 60 Han (21.3%) and 187 (59.9%) Uygur were KSHV seropositive. Again, KSHV infection was correlated with advancing age ($P = 0.047$) and education level ($P = 0.003$). The stationary population showed higher KSHV seroprevalence (44.4%) than the immigrated population (25.3%). No clear correlation was observed between KSHV infection and gender in either Urumqi or Kashgar participants.

### Peripheral blood DNA positive rate and risk factors in Urumqi region

All peripheral blood DNA samples from Urumqi were analyzed using nested PCR for the presence of KSHV DNA based on primers for the *ORF26* and *gB* genes. Reaction products of *ORF26* and *gB* by nested PCR in the second round showed that the target gene fragments were amplified in a position-specific manner, and the product size was 174 bp and 383 bp, respectively (Supplemenaty Figure S1A, 1B). Samples scored positive for KSHV DNA detection were positive for amplification of both the *ORF26* and *gB* genes. Table 3 presents the KSHV DNA positive rate associations with ethnicity, gender, age, and education, and stratified by population status (stationary or immigrated). There were 26.9% (88/327) of Uygur participants and 17.4% (65/373) of Han participants whose peripheral blood DNA showed KSHV-positive results, which indicated a significant difference ($P = 0.002$). Among all participants, the KSHV DNA positive rate increased significantly with age from 18.9% in those aged < 50 years to 25.7% in those aged ≥ 50 years ($P = 0.03$). Furthermore, there was a significant difference in the KSHV DNA positive rate with respect to population status ($P = 0.018$), from 17.1% in the immigrated population to 24.7% in the stationary population. A correlation of borderline significance was detected between KSHV DNA positive rate and education level ($P = 0.052$), while no associations were observed between KSHV infection and gender.

### KSHV neutralizing antibody prevalence and titers

Given the high prevalence of KSHV infection in people of both Uygur and Han ethnicity in Xinjiang, and the high KSHV antibody titer in those of Uygur ethnicity, we investigated the prevalence of neutralizing antibodies in all KSHV-positive participants in Urumqi. We found that 17/132 (12.9%) seropositive Uygur participants had detectable levels of neutralizing antibodies. In contrast, only 5/116 (4.3%) KSHV-seropositive Han participants had detectable neutralizing antibody (Figure 2A). Since there was a significant difference ($P = 0.018$) in the

Table 2. The associations of KSHV seroprevalence with population characteristics stratified by geographic region from Xinjiang, China

| Characteristic | Cases (n) | Urumqi | | $\chi^2$ | *P* | Cases (n) | Kashgar | | $\chi^2$ | *P* |
|---|---|---|---|---|---|---|---|---|---|---|
| | | KSHV + (%) | KSHV - (%) | | | | KSHV + (%) | KSHV - (%) | | |
| **Ethnicity** | | | | | | | | | | |
| Han | 373 | 116 (31.1) | 257 (68.9) | 6.542 | 0.011 | 282 | 60 (21.3) | 222 (78.7) | 91.132 | 0.000 |
| Uygur | 327 | 132 (40.4) | 195 (59.6) | | | 312 | 187 (59.9) | 125 (40.1) | | |
| **Gender** | | | | | | | | | | |
| Male | 364 | 136 (37.4) | 228 (62.6) | 1.240 | 0.265 | 244 | 105 (43.0) | 139 (57.0) | 0.359 | 0.549 |
| Female | 336 | 112 (33.3) | 224 (66.7) | | | 350 | 142 (40.6) | 208 (59.4) | | |
| **Age** | | | | | | | | | | |
| < 50 | 397 | 123 (31.0) | 274 (69.0) | 7.926 | 0.005 | 286 | 107 (37.4) | 179 (62.6) | 3.948 | 0.047 |
| ≥ 50 | 303 | 125 (41.3) | 178 (58.7) | | | 308 | 140 (45.5) | 168 (54.5) | | |
| **Education** | | | | | | | | | | |
| Elementary or below | 273 | 109 (39.9) | 164 (60.1) | 3.958 | 0.047 | 330 | 155 (47.0) | 175 (53.0) | 8.871 | 0.003 |
| Junior high or beyond | 427 | 139 (32.6) | 288 (67.4) | | | 264 | 92 (34.8) | 172 (65.2) | | |
| **Population** | | | | | | | | | | |
| Stationary population | 437 | 168 (38.4) | 269 (61.6) | 4.623 | 0.032 | 507 | 225 (44.4) | 282 (55.6) | 11.142 | 0.001 |
| Immigrated population | 263 | 80 (30.4) | 183 (69.6) | | | 87 | 22 (25.3) | 65 (74.7) | | |

prevalence of neutralizing antibodies between samples from individuals of Uygur and Han ethnicity, all neutralizing antibody positive samples were further titrated to investigate whether there were differences in the neutralizing antibody titers within these groups. Figure 2B compares the GMT of neutralizing antibodies of all samples that had detectable neutralizing antibodies between Han and Uygur participants. The result clearly demonstrated that the GMT of neutralizing antibodies in those of Uygur ethnicity was significantly higher than that in those of Han ethnicity ($P = 0.026$).

Figure 2. Prevalence and titer of KSHV neutralizing antibodies in Han and Uygur groups in the study population in Urumqi, Xinjiang. (A) Percentage prevalence of neutralizing antibodies in individuals of Han and Uygur ethnicity. (B) KSHV neutralizing antibody titers ($log_2$ transformed) in samples from Han and Uygur participants. GMT of neutralizing antibodies in each group is also marked.

### DISCUSSION

This is the first study to our knowledge conducted to compare KSHV seroprevalence and antibody titers between people of Uygur and Han ethnicity in the Urumqi and Kashgar regions of Xinjiang, China. Moreover, it is also the first comparison between the prevalence and titers of neutralizing antibodies in plasma samples from Uygur and Han individuals from an endemic area. Xinjiang has a unique mix of various ethnicities and has been reported to be an endemic area for KS and KSHV infection. He *et al.* (2007) reported that more than 95% of KS cases were observed in minority groups, particularly of Uygur ethnicity. Xinjiang's vast territory, in the center of the Eurasian continent, is inhabited by minorities with distinctive languages, cultures, religions, and lifestyles, unique diets, and different genetic backgrounds. In

Table 3. The associations of KSHV DNA positive rate with population characteristics in Urumqi region of Xinjiang.

| Characteristic | Cases (n) | KSHV + (%) | KSHV - (%) | $\chi^2$ | *P* |
|---|---|---|---|---|---|
| **Ethnicity** | | | | | |
| Han | 373 | 65 (17.4) | 308 (82.6) | 9.178 | 0.002 |
| Uygur | 327 | 88 (26.9) | 239 (73.1) | | |
| **Gender** | | | | | |
| Male | 364 | 72 (19.8) | 292 (80.2) | 1.915 | 0.166 |
| Female | 336 | 81 (24.1) | 255 (75.9) | | |
| **Age** | | | | | |
| < 50 | 397 | 75 (18.9) | 322 (81.1) | 4.722 | 0.030 |
| ≥ 50 | 303 | 78 (25.7) | 225 (74.3) | | |
| **Education** | | | | | |
| Elementary or below | 354 | 88 (24.9) | 266 (75.1) | 3.778 | 0.052 |
| Junior high or beyond | 346 | 65 (18.8) | 281 (81.2) | | |
| **Population** | | | | | |
| Stationary population | 437 | 108 (24.7) | 329 (75.3) | 5.558 | 0.018 |
| Immigrated population | 263 | 45 (17.1) | 218 (82.9) | | |

addition, the minority populations in the region are relatively less mobile comparing with Han ethnicity. Several studies have investigated KSHV seroprevalence in people of Uygur or Han ethnicity in Xinjiang, but none have dealt with seroprevalence of KSHV, peripheral blood KSHV DNA positive rate, and the prevalence of neutralizing antibodies, and identified risk factors associated with KSHV prevalence among populations of Uygur and Han ethnicity from different regions.

In this study, the overall KSHV seroprevalence and the endpoint titer of Uygur participants were significantly higher than those of Han participants in both Urumqi and Kashgar regions. Previous studies have shown a high KSHV seroprevalence in Xinjiang, in the range of 12.5% to 48.0%, depending on differences in study populations (Fu et al., 2009; Du et al., 2000; Wang et al., 2010; He et al., 2007). The seroprevalence of KSHV among the general Uygur population from South and North Xinjiang was 23.1% and 25.9%, respectively (Wang et al., 2011). Compared with the Han population in mainland China, which has a very low serum-positive rate, the significantly increased rate in the Han population in Xinjiang also presents a thought-provoking question. This study mainly concentrated on the Chinese Han population that immigrated to Xinjiang during the 1950s to 1970s. Due to historical background and traditional moral values, those of Han nationality conform very strictly to single-partner sexual relationships. Moreover, because of religious differences between Uygur and Han populations, interracial marriage, sexual relations, and drug needle sharing between these groups are extremely rare. Therefore, the possibility of sexual contact is unlikely. Is it possible that the Han population itself belongs to a high-risk group for high KSHV positive rate among the Han population in Xinjiang? This possibility is unlikely, considering the very low positive rate among the Han population in mainland China. At the time the Han population immigrated to Xinjiang, both health care and living conditions were primitive and difficult in Xinjiang, and there was a high likelihood of horizontal transmission.

In comparison with KSHV seroprevalence, the positive rate of peripheral blood DNA was relatively low. KSHV belongs to the gamma herpesvirus family and is similar to other herpesviruses. After infection, it can produce two different life cycles, latent infection and lytic replication, and can reside in the host for a lifetime. After the initial infection, the viral genome continues to exist in the nucleus in the form of covalent rings. The virus is first evading host immune clearance. Furthermore, when the body is exposed to various adverse stresses, the decreased immune status allows activation of the latent virus and it enters the state of lytic replication. This then produces a large number of virus particles, which further infect B lymphocytes and play an extremely important role in induction of carcinogenesis. Therefore, the detection rate of KSHV DNA in peripheral blood mononuclear cells (PBMC) is relatively low. Considering the detection rate of the PBMC genome (about 1.33%–22.5%), both domestically and internationally, this provides further evidence that Xinjiang is a high-prevalence area for KSHV. As previously reported, KSHV DNA positive rates were 4.5% and 22.5% among donors in Tanzania (Enbom et al., 2002) and Central Africa (Belec et al., 1998). According to another report, the detection rate of KSHV DNA in 59 blood donors in Italy was 9% (Bigoni et al., 1996). The positive KSHV DNA rate among 230 healthy blood donors in northeast China was 7.8% (Wang et al., 2002). It is worthwhile noting that there is no direct correlation between the positive rate of serum and the positive rate of DNA in peripheral blood. In other words, the positive rate of serum or DNA reflects the KSHV infection in different groups. The positive rate of serum is mainly detected by serum antibody, while the DNA test can be regarded as antigen expression. In one study, it was reported that the detection rate of KSHV DNA in PBMC was 16.5% in 158 healthy KSHV-seropositive adults (Brown et al., 2005). In another work, the KSHV DNA in PBMC was detected only in one person out of 12 KSHV-seropositive healthy individuals (Vitale et al., 2000).

Further analysis of the risk factors associated with KSHV indicated that KSHV infection was associated with advancing age, low education level, and stationary population status. These results are consistent with those of several other studies, which described an increase in seroprevalence of KSHV with age in different populations (Baillargeon et al., 2001; Zavitsanou et al., 2007; 2010). This correlation may be related to the natural aging process, and hypoimmunity may be a promoting agent for KSHV infection. In this study, the educational level of the population was relatively low, especially in the Kashgar area, where nearly half of the people had an education level below junior high school. Understandably, general knowledge and awareness about health issues is far more limited in the population in this region. Therefore, attaining a higher education level is a protection factor for KSHV infection. Furthermore, the mobility of the population in Xinjiang region is relatively low. The stationary population is mostly of Uygur ethnicity, and these residents are relatively concentrated. To a large extent, the traditional lifestyle and behavior of the Uygur people has remained unchanged over the past two decades. In addition, the lifestyle and eating habits of some Han populations who have lived in Xinjiang for a long time may have become adversely affected, with quality of life and education level reflecting their low socioeconomic status. Such conditions might have facilitated the transmission of KSHV infection.

The neutralizing antibody response is known to play an important role in preventing viral infection (Yoshida et al., 2002) and may be protective against the development of KS (Kimball et al., 2004). Our finding that Uygur participants had a high prevalence and increased titers of neutralizing antibodies should not be interpreted to mean that neutralizing antibodies are not protective in the case of KSHV infection. It is quite possible that neutralizing antibodies have very different roles during primary KSHV infection and later on during persistent long-term infection. In chronically infected KS patients, neutralizing antibodies may have a role limited only to controlling the spread of KSHV. However, during primary infection, neutralizing antibodies can prevent KSHV infection if they are present at the time of exposure, as would be the case if elicited by a vaccine prior to primary infection. A prophylactic vaccine that can strongly induce neutralizing antibodies could be useful to prevent infection in KSHV endemic populations. In addition, longitudinal studies are needed to investigate the association between the presence of neutralizing antibodies and the development of KS in this population.

In summary, we identified a high seroprevalence of KSHV and high peripheral blood DNA infection among the general Uygur and Han populations in both Urumqi and Kashgar regions in Xinjiang. Through analysis of the potential correlation between KSHV prevalence and risk factors in endemic areas of Xinjiang, it can be determined that advanced age, low education level, and stationary population status likely affect KSHV infection rates. KSHV-positive Uygur participants showed higher prevalence of neutralizing antibodies and neutralizing antibody titers than KSHV-positive Han participants. Further studies on KSHV prevalence and more extensive risk factor analysis, such as living arrangements, hygiene conditions, and food-sharing practices, are needed to explore the epidemiology of KSHV infection in this population.

### ACKNOWLEDGMENTS

This study was supported by research grants from the National Natural Science Foundation of China (U1603117; 81560473; 81560442), Doctoral Foundation and Technology Research and Achievements Transformation Program of Xinjiang production and Construction Corps (2014BB021; 2015AD003), the United States National Institutes of Health Fogarty International Center (D43 TW001492), NCI (CA75903) and NCRR COBRE (RR15635) to C. Wood; Y. Zeng was a Fogarty Scholar.

### COMPLIANCE WITH ETHICS GUIDELINES

The authors declared that there is no conflict of interest. The study was approved by the Institutional Review Board of the University of Nebraska-Lincoln and the ethics board of Shihezi University, and written informed consent was obtained from all participants.

### AUTHOR CONTRIBUTIONS

YZ and TJZ designed the experiments. JZ, YY, MC and YZ carried out the experiments. ZJS, LLH and JZ acquired the clinical samples, ZQL and TJZ analyzed the data. CW provided scientific input. JZ and YZ wrote the manuscript. All authors read and approved the final manuscript.

### OPEN ACCESS

This article is distributed under the terms of the Creative Commons Attribution 4.0 International License (https://creativecommons.org/licenses/by/4.0/), which permits unrestricted use, distribution, and reproduction in any medium, provided you give appropriate credit to the original author(s) and the source, provide a link to the Creative Commons license, and indicate if changes were made.

Supplementary figures and tables are available on the websites of *Virologica Sinica*: www.virosin.org; link.springer.com/journal/12250.

## Electronic supplementary material

Prevalence of Kaposi’s sarcoma-associated herpesvirus in Uygur and Han populations from the Urumqi and Kashgar regions of Xinjiang, China
