# Supplementary material for: Prevalence of Kaposi’s sarcoma-associated herpesvirus in Uygur and Han populations from the Urumqi and Kashgar regions of Xinjiang, China
Source: Virol Sin. 2017 Oct 25;32(5):396–403. doi: 10.1007/s12250-017-4049-9 (PMC6704202; doi:10.1007/s12250-017-4049-9)
Supplement: Supplementary file 1 — Prevalence of Kaposi’s sarcoma-associated herpesvirus in Uygur and Han populations from the Urumqi and Kashgar regions of Xinjiang, China [file 12250_2017_4049_MOESM1_ESM.pdf]

Electronic Supplementary Material

Prevalence of Kaposi's sarcoma-associated herpesvirus in Uygur and Han populations from the Urumqi and Kashgar regions of Xinjiang, China

Jun Zheng[1,2#], Yang Yang[1#], Meng Cui[1#], Zhan-Jun Shu[3], Li-Li Han[4], Zhen-Qiu Liu[5], Charles Wood[6], Tiejun Zhang[5✉], Yan Zeng[1✉]

1. Key Laboratory of Xinjiang Endemic and Ethnic Disease & Department of Biochemistry, School of Medicine, Shihezi University, Shihezi 832000, China
2. Department of Stomatology, The First Affiliated Hospital, School of Medicine, Shihezi University, Shihezi 832000, China
3. Division of AIDS Research, National Traditional Chinese Medicine Clinical Research Bases in Xinjiang , Urumqi 830000, China
4. Department of Gynecology, Xinjiang Uygur Autonomous Region People's Hospital, Urumqi 830001, China
5. Department of Epidemiology, School of Public Health, Fudan University, Shanghai 200032, China
6. Nebraska Center of Virology and the School of Biological Sciences, University of Nebraska-Lincoln, Lincoln 68583, USA

Supporting information to DOI: 10.1007/s12250-017-4049-9

Table S1. Primer sequences

Primers		Sequence
β-actin	Forward Primer (5′–3′)	TTCTACAATGAGCTGCGTGT
	Reverse Primer (5′–3′)	GCCAGACAGCACTGTGTTGG
ORF26 (First Round)	Forward Primer (5′–3′)	AGCCGAAAGATTCCACCAT
	Reverse Primer (5′–3′)	TCCGTGTTGTCTACGTCCAG
ORF26 (Second Round)	Forward Primer (5′–3′)	CGAATCCAACGGATTTGACCTC
	Reverse Primer (5′–3′)	CCCATAAATGACACATTGGTGGTA
gB (First Round)	Forward Primer (5′–3′)	GACCTGTACGCCCTTCTGTAC
	Reverse Primer (5′–3′)	ATAGAGTGGCACGGGTCTC
gB (Second Round)	Forward Primer (5′–3′)	GCCACCCTGGGGACTGTCAT
	Reverse Primer (5′–3′)	TTGGTGATGGCGGACTCTGTC

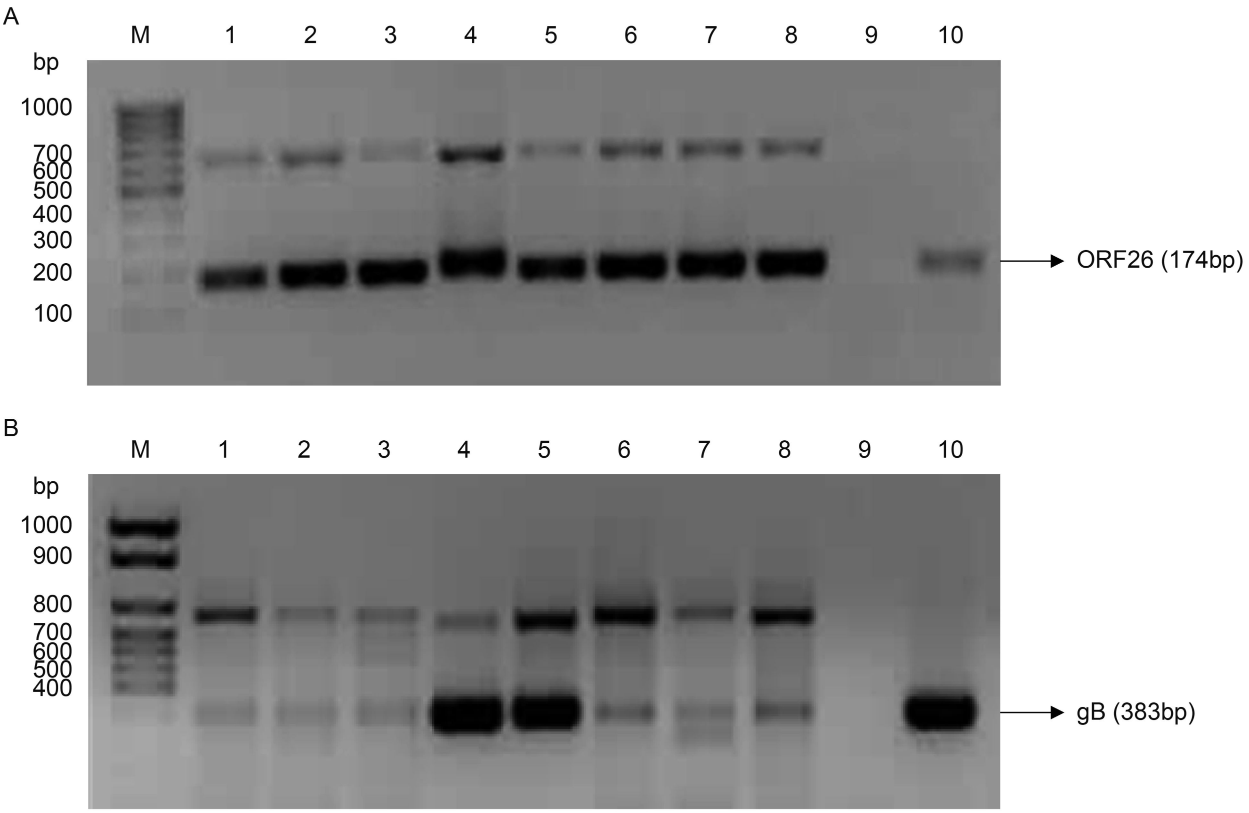

Figure S1. Reaction products of *ORF26* and *gB* by nested PCR in the study population in Urumqi, Xinjiang. (A) Reaction products of *ORF26* by nested PCR in the second round. (B) Reaction products of *gB* by nested PCR in the second round. M: DNA marker; 1–8: reaction products of KSHV serum positive samples; 9: negative control (double-distilled H_2O); 10: positive control (DNA of KSHV).
